# Functional network antagonism and consciousness

**DOI:** 10.1162/netn_a_00244

**Published:** 2022-10-01

**Authors:** Athena Demertzi, Aaron Kucyi, Adrián Ponce-Alvarez, Georgios A. Keliris, Susan Whitfield-Gabrieli, Gustavo Deco

**Affiliations:** Physiology of Cognition, GIGA Consciousness Research Unit, GIGA Institute (B34), Sart Tilman, University of Liège, Liège, Belgium; Psychology and Neuroscience of Cognition (PsyNCog), Faculty of Psychology, Logopedics and Educational Sciences, Sart Tilman, University of Liège, Liège, Belgium; GIGA-CRC In Vivo Imaging, Sart Tilman, University of Liège, Liège, Belgium; Fund for Scientific Research, FNRS, Bruxelles, Belgium; Department of Psychology, Northeastern University, Boston, MA, USA; Center for Brain and Cognition, Computational Neuroscience Group, Department of Information and Communication Technologies, Universitat Pompeu Fabra, Barcelona, Spain; Bio-Imaging Lab, Department of Biomedical Sciences, University of Antwerp, Wilrijk, Belgium; Northeastern University Biomedical Imaging Center (NUBIC), Northeastern University Interdisciplinary Science and Engineering Complex (ISEC), Boston, MA, USA; Institució Catalana de la Recerca i Estudis Avançats (ICREA), Barcelona, Spain; Department of Neuropsychology, Max Planck Institute for Human Cognitive and Brain Sciences, Leipzig, Germany; Turner Institute for Brain and Mental Health, School of Psychological Sciences, and Monash Biomedical Imaging, Monash University, Clayton, Melbourne, VIC, Australia

**Keywords:** Anticorrelations, Integration, Segregation, Consciousness, Neural inhibition, Functional connectivity

## Abstract

Spontaneous brain activity changes across states of consciousness. A particular consciousness-mediated configuration is the anticorrelations between the default mode network and other brain regions. What this antagonistic organization implies about consciousness to date remains inconclusive. In this Perspective Article, we propose that anticorrelations are the physiological expression of the concept of segregation, namely the brain’s capacity to show selectivity in the way areas will be functionally connected. We postulate that this effect is mediated by the process of neural inhibition, by regulating global and local inhibitory activity. While recognizing that this effect can also result from other mechanisms, neural inhibition helps the understanding of how network metastability is affected after disrupting local and global neural balance. In combination with relevant theories of consciousness, we suggest that anticorrelations are a physiological prior that can work as a marker of preserved consciousness. We predict that if the brain is not in a state to host anticorrelations, then most likely the individual does not entertain subjective experience. We believe that this link between anticorrelations and the underlying physiology will help not only to comprehend how consciousness happens, but also conceptualize effective interventions for treating consciousness disorders in which anticorrelations seem particularly affected.

## Author Summary

The fMRI resting paradigm can quantify brain function by surpassing communication and sophisticated setups, hence helping to infer consciousness in individuals who are unable to communicate with their environment. A particular consciousness-mediated rsfMRI configuration is that of functional anticorrelations, that is, the antagonistic relationship between a specific set of brain regions. We suggest that anticorrelations are a key physiological prior, without which consciousness cannot be supported, because the brain cannot segregate how regions get connected. We postulate that segregation is possible thanks to neural inhibition, by regulating global and local inhibitory activity. We believe that the link between anticorrelations and the underlying physiology can help not only to comprehend how consciousness happens, but also conceptualize effective interventions for treating its disorders.

## INTRODUCTION

Individuals during sleep, anesthesia, and in disorders of consciousness are unable to communicate intentionally with the environment. Consequently, their mental state needs to be inferred by means of meaningful proxies. The fMRI resting paradigm has been a great asset to that matter, as it quantifies brain function by surpassing the need for communication of experience or behavioral output (Zhang et al., 2021). Overall, studies in such states of consciousness point to lesser functional connectivity (FC) between regions that are within the same “network,” in that they show positive FC during wakefulness (Heine et al., 2012). The positive coupling between brain regions, especially those of the default mode network (DMN), was shown to enable fast and accurate performance during higher order cognitive tasks concerning, for example, executive function (Shine et al., 2016) or working memory (Cohen & D’Esposito, 2016). Such integrative profiles reduce dramatically in states of unconsciousness—yet within-network FC persists and does not entirely disappear (Boveroux et al., 2010; Di Perri et al., 2016). Therefore, DMN correlations might be more about shaping connectivity interactions rather than reflecting conscious mental activity (Boly et al., 2008).

A rather consciousness-sensitive connectivity profile is that of functional anticorrelations, that is, the negative FC that some regions show with the DMN. Depending on the state of consciousness anticorrelations reduce in intensity, like after sleep deprivation (De Havas et al., 2012; Yeo et al., 2015), in slow wave sleep and REM (Chow et al., 2013), hypnosis (Demertzi et al., 2011), and deep sedation (Luppi et al., 2019). Or they are undetectable, like in deep anesthesia (Boveroux et al., 2010), and unresponsive brain-damaged patients (Di Perri et al., 2016; Threlkeld et al., 2018). Importantly, anticorrelations’ FC recovers during the immediate postanesthetic period (Nir et al., 2020) and after emergence from a disorder of consciousness (Di Perri et al., 2016; Threlkeld et al., 2018). Within the state of typical wakefulness, the presence of anticorrelations was shown to contributes to cognitive function (J. B. Keller et al., 2015; Vanhaudenhuyse et al., 2011), with greater intensity leading to better within-subject (e.g., Kucyi et al., 2017) and between-subject performance (e.g., Spreng et al., 2010). Also, anticorrelations seem to contribute to life span, starting weak in children, strengthening during adolescence, ending up fully anticorrelated in young adulthood (Chai et al., 2014), and getting selectively decreased during healthy aging (J. B. Keller et al., 2015). Considering that anticorrelations are implicated in cognition and consciousness, an emerging question is what this antagonistic configuration implies about the brain’s physiology and conscious experience. To our knowledge, no such formulation has been suggested yet. To address this, we discuss conceptual and methodological debates around anticorrelations, and, by tackling their physiological underpinnings, we postulate a mechanistic link between micro- and macrocircuitry, which may explain the function of anticorrelations in the context of conscious experience.

## FUNCTIONAL ANTICORRELATIONS CONTAIN MEANINGFUL NEURAL ACTIVITY

Anticorrelations refer to brain regions showing negative FC in contrast to the positive FC within intrinsic networks, such as the DMN. The regions showing negative FC with the DMN concern primarily the intraparietal sulcus, the frontal eye fields, and the middle temporal + area (Fox et al., 2005). Historically, the areas showing anticorrelations were coined as “task-positive” in contrast to a “task-negative” DMN (Fox et al., 2005). This connotation was given to highlight, respectively, activations and deactivations exhibited by these systems during task performance, initially measured with positron emission tomography, and later confirmed by fMRI (Raichle & Mintun, 2006). It has been proposed, however, that the dichotomization between “task-positive” and “task-negative” might be misleading because it insinuates that the DMN is not engaged actively in cognitive processes (Spreng, 2012). As the DMN indeed collaborates with other task-related areas (Elton & Gao, 2015) and networks (Spreng, 2012) to promote cognitive performance and mental flexibility (Spreng et al., 2014), we will here preferentially utilize the term *anticorrelations*.

The discussion about anticorrelations very often goes hand in hand with the methodological debate about correcting or not for the brain’s global signal (GS) during fMRI data preprocessing. The GS can be obtained by averaging the resting-state time courses over the entire brain (Desjardins et al., 2001). This whole-brain averaging implies the possibility that nonneuronal sources can contribute to the GS along with neural signal. As most functional connectivity studies are interested in identifying the neural counterparts of a task or a condition, this implies that GS needs to be accounted for. GS correction can happen via linear regression, subtraction, or normalization (T. T. Liu et al., 2017). Such a process, however, can lead to systematically shifting the distribution of correlation values in the negative direction (Anderson et al., 2011; Murphy et al., 2009) and, so, anticorrelations emerge. This implies that anticorrelations are a matter of mathematical treatment, spurious, and not neuronally meaningful. To date, however, there is support both for the nonneuronal and the neuronal significance of the GS. On the one hand, the GS is shown to reflect fMRI nuisance sources such as motion, scanner artifacts, respiration (Power et al., 2017), cardiac rate (Chang & Glover, 2010), and vascular activity (Colenbier et al., 2020; Zhu et al., 2015). On the other hand, GS is considered to have a neuronal counterpart (Schölvinck et al., 2010) that promotes behavior (Li et al., 2019), it was shown to correlate with spontaneous fluctuations in the local field potentials as measured with implanted electrodes in monkeys (Schölvinck et al., 2010), and it was associated with vigilance (Wen & Liu, 2016; Wong et al., 2013) and arousal (X. Liu et al., 2018) as measured with EEG in humans. Together, the debate about whether to employ GS correction as a preprocessing step or not remains unresolved, while the choice can be driven by the research question at hand (Murphy & Fox, 2017; Uddin, 2017).

Generally, we align with the view that the spontaneous anticorrelations are not mere artifacts and that they actually reflect neural activity. This is after considering that GS correction does not preferentially affect only systems exhibiting positive correlations but also those which show anticorrelations in the first place (Fox et al., 2009). Anticorrelations between the DMN and the executive attention system can also be found using independent component analysis (without GS correction), suggesting that the anticorrelations are not merely a mathematical issue. Also, anticorrelations are shown to increase after caffeine intake, pointing to their physiological dynamism (Wong et al., 2013). The anticorrelations between networks homologous to DMN and the dorsal attention network (DAN) are also observed in rodents, dogs, and nonhuman primates, confirming interspecies consistency (Belloy et al., 2018a; Gozzi & Schwarz, 2016; Hutchison & Everling, 2012; Szabó et al., 2019). Furthermore, the strength of anticorrelations is shown to be predictive of disease phenotype (Adhikari et al., 2021; Belloy et al., 2018b; Sripada et al., 2014) and is able to change by means of sensory stimulation, attention, and neuromodulation (Hinz et al., 2019; Peeters et al., 2020). Finally, the existence of spontaneous anticorrelated networks is evidenced in computational simulations in monkey and human brains (Deco et al., 2009), as well as in neurophysiological studies. By means of simultaneous scalp EEG and fMRI in humans, a temporal relationship has been found between increased alpha power and greater DMN-DAN anticorrelations (Chang et al., 2013), highlighting their cognitive relevance. More direct evidence comes from intracranial recordings of local field potentials (LFPs). In cats, simultaneous recordings of unit activity and LFPs showed that, when attentional demands increased, LFP power in task-on (DAN-homologue) regions augmented and task-off (DMN-homologue) regions decreased (Popa et al., 2009). Further human intracranial EEG evidence suggests that during wakeful rest, areas of these networks exhibited anticorrelated slow fluctuations of high gamma power (C. J. Keller et al., 2013), which was correlated with neuronal firing rates (Manning et al., 2009). Also, high gamma power evoked by tasks had opposing patterns in the DMN and antagonistic networks as measured by intracranial EEG (Ossandón et al., 2011; Ramot et al., 2012). Finally, intracranial EEG supports that spontaneous, transient increases in high gamma (a proxy for neuronal spiking) regularly arose within major nodes of the DMN versus DAN/salience networks and were time-locked to cognitive and physiological events (Daitch & Parvizi, 2018; Kucyi & Parvizi, 2020). Collectively, observational and interventional studies in humans and animals support that anticorrelations have a physiological and neuronal importance.

## NEURAL INHIBITION MEDIATES THE FORMULATION OF ANTICORRELATIONS

We postulate that anticorrelations may be emerging thanks to the process of neural inhibition. Neural inhibition is a pivotal mechanism for the brain to sustain balanced cortical activity (Isaacson & Scanziani, 2011). This is done by the orchestrated coordination between excitatory pyramidal spiking neurons occupying 70%–80% of the cortex and the remaining inhibitory nonpyramidal cells (DeFelipe & Fariñas, 1992), such that for every five excitatory synapses there is approximately one inhibitory (Beaulieu & Colonnier, 1985). Excitation and inhibition happen in a balanced way, leaving it unlikely to observe an increase in one without observing an increase in the other; otherwise, no cell would reach firing threshold (Scannell & Young, 1999). The recruitment of GABAergic inhibitory interneurons via the thalamus, corticocortical, or other excitation pathways has been shown to assist cognition and motor behavior (Swanson & Maffei, 2019). Also, it is via this synchronous activation of excitatory and inhibitory activity that anticorrelated cortical network activity emerges (Arthurs & Boniface, 2002; Logothetis, 2008).

**Figure 1.**
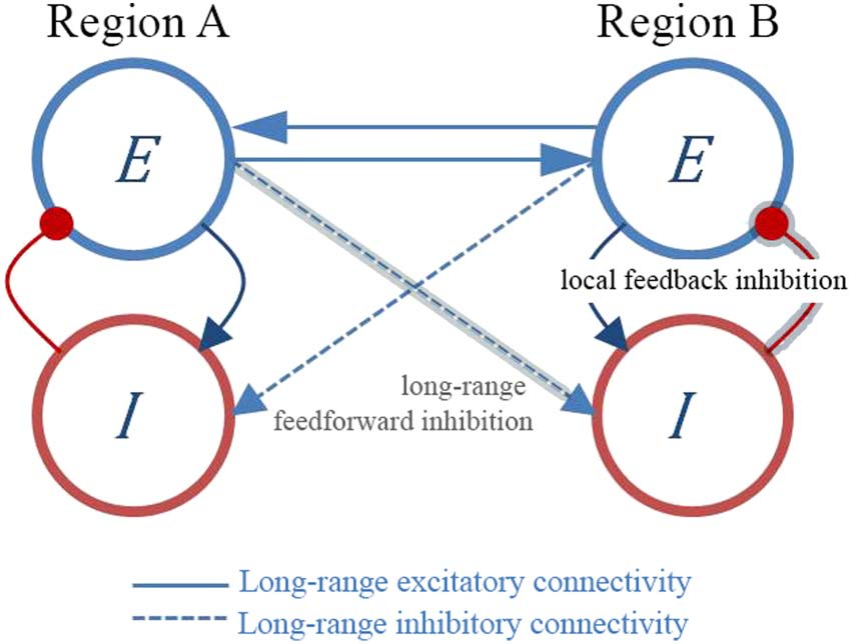
Functional anticorrelations might emerge as a result of local and long-range inhibitory activity. Brain regions are modeled by interconnected populations of excitatory (E) and inhibitory (I) neurons. Long-range excitatory connections from brain region A to brain region B can target the excitatory population (E → E, solid blue) or the inhibitory population (E → I, dashed blue). The level of inhibition in region B depends i) on the local E-I loop (local feedback inhibition) and ii) on the excitatory inputs from region A to the inhibitory neurons in region B, which in turn connect locally to the excitatory cells (long-range feedforward inhibition, gray highlighted path). With this model, we postulate that neural inhibition assists the emergence of the anticorrelations not by direct internetwork, interareal inhibition (of note, most interareal connections around the brain are glutamatergic). Rather, anticorrelations appear indirectly after disrupting both local and global balance between excitation and inhibition, eventually affecting network metastability.

Considering this mechanism in relation to anticorrelations, we do not claim that neural inhibition directly promotes anticorrelated patterns, that is, by having networks straightly inhibiting one another (of note, most interareal connections are glutamatergic/excitatory). Nor do we claim that neural inhibition always leads to anticorrelated profiles. Rather, we suggest that neural inhibition mediates the rise of the anticorrelations indirectly, by breaking the local neural balance which affects network metastability and which eventually permits anticorrelations to appear. More particularly, current computational whole-brain models assume that distant brain regions establish connections between their corresponding excitatory neural populations (Figure 1, E-E blue solid line connectivity). The resulting local increase of excitation produces an increase of inhibition through the local E-I loop (feedback inhibition). Apart from the interareal excitatory activity, one brain region (A) can also effectively inhibit the activity of a distal brain region (B), by A targeting B’s inhibitory interneurons (Figure 1, E-I blue dashed line connectivity), which in turn, locally connect to the pyramidal cells (Figure 1, I-E red connector; i.e., feedforward inhibition; Isaacson & Scanziani, 2011). However, little attention has been paid to this indirect long-range inhibition in modeling studies and how specifically feedforward inhibition interacts with the local excitation-inhibition (E/I) ratio. Although previous theoretical work has separately examined the effect of regulating the E/I ratio through feedback inhibition or feedforward inhibition in whole-brain models (Deco et al., 2014), a model that takes both effects into account has not been studied yet. Local heterogenous feedback inhibition, in particular, signifies that the excitability of local population activity is achieved by variably determining each region’s gain response function. Recent studies using heterogeneous and homogeneous whole-brain modeling have mechanistically shown that, besides increasing the level of fitting of the empirical data, an increase in ignition was observed (Deco et al., 2021). Based on this evidence, one could speculate that ignition is related to the presence of regional heterogeneity. Although the type of heterogeneity that is the most relevant for ignition remains open, one can nevertheless say that thanks to heterogeneity in general, ignition-like dynamics may happen (Deco et al., 2021), which are thought to support conscious experience (see next section).

Critically, it has been shown that, when feedback projections and randomization of the connectivity weights were removed, dynamic network behavior was abolished, suggesting that feedback connectivity and heterogeneity in connection strength gives rise to ignition-like activity (Joglekar et al., 2018). We here hypothesize that the generation of the anticorrelations can be a matter of both inhibitory effects (local feedback inhibition, long-range feedforward inhibition), which will eventually affect network metastability, allowing the emergence of the anticorrelations. To date, the behavior of this model remains to be theoretically studied and empirically supported.

## THE ANTICORRELATIONS’ SIGNIFICANCE TO CONSCIOUSNESS

On our quest to tackle the meaning of the anticorrelations to consciousness we lean toward theoretical frameworks in order to see how they embrace the mechanism of inhibition. We notice that especially the global neuronal workspace theory (GNWT; Dehaene et al., 2003) and the integrated information theory (IIT; Oizumi et al., 2014) are two such theories that utilize inhibition when accounting for consciousness in their models.

The GNWT describes how reportable experience happens. The theory proposes a model with various local processors that are all linked at a central executive. Its model suggests to view the local processors as specialized modular cortical areas that process specific perceptual, motor, memory, and evaluative information. The central executive (composed of widely distributed excitatory neurons) can be considered as a second computational space, which forms reciprocal connections to specific processor neurons. Based on this architecture, at any moment, a piece of information within one or several processors can be selected, amplified, and broadcasted to all other processors, thus rendering it consciously accessible and available for reporting. This global broadcasting happens thanks to ignition (Dehaene et al., 2003; Mashour et al., 2020). Ignition is characterized by the sudden, coherent, and exclusive activation of a subset of workspace neurons that codes the current conscious content, while the remainder of the workspace neurons remain inhibited. Regardless of how ignition may be triggered, simulations show that if feedforward connections are carefully balanced by local inhibitory influences, incoming stimuli elicit a stable cascade of activity characterized by a late and sudden ignition. In other words, ignition of a global neural state coding for particular conscious content leads to the active inhibition of other potential contents (as we also showed before; Vanhaudenhuyse et al., 2011).

The IIT begins the quest of consciousness by experience itself. The theory first identifies the experience’s essential properties (axioms) and then infers what kind of properties a physical system must have in order to account for these properties (postulates). The postulates specify which conditions (such as neurons and their connections) must be satisfied by physical mechanisms to account for the phenomenology of experience. To generate consciousness, a physical system must be able to discriminate among a large repertoire of states (information), and it must be doing so as a single system, therefore not decomposable into a collection of causally independent parts (integration). For integrated information to be high, a system must be connected in such a way that information is generated by causal interactions among its parts, rather than within them. In balanced states, the corticothalamic system is a fine example of a functionally integrated and specialized network, able to generate high values of integrated information peaks that are characterized by a complex spatiotemporal pattern of signal propagations in different directions (feedforward, feedback, contralateral) and with variant weights, including inhibitory. In unbalanced states, like those of low arousal, the cortical neurons are inactivated due to the neuronal bistability of their membrane potential or active inhibition, and thus cannot specify a conceptual structure. In that case, the system then collapses, leading to low values of integrated information, and hence diminished conscious experience.

If we try to link the inhibitory mechanisms described in these models with functional anticorrelations, we can observe that these promote the notion of functional segregation. In that respect, anticorrelations can be considered as an FC segregated profile, which mediates various states of consciousness. In the past, Fox et al. (2005) similarly suggested that “while correlations may serve as an integrative role in combining neuronal activity subserving similar goals or representations, anticorrelations may serve as a differentiating role segregating neuronal processes, subserving opposite goals or competing representations” (Fox et al., 2005, p. 9677). This view on segregation refers to the ability of a system to distinguish information into distinct modules that can perform specialized local computations (Shannon, 1948). This definition may slightly deviate from how graph theory considers segregation, that is, close to the notion of modularity and as a measure of the relative strength between a graph’s nodes (Sporns, 2013). In that respect, inhibition as described in the GNWT can be viewed as the segregative processes that hinder widespread FC, leading to negative functional correlations. Similarly, differentiation as described in the IIT can be similarly considered as parallel to the processes of segregation, also expressed as anticorrelated FC.

Taken together, our view is that anticorrelations are the physiological expression of segregation, and we propose that neural inhibition is the mediating link. Our stance, of course, does not prevent other measures from being linked to unconsciousness. Indeed, low values in sample entropy (which quantifies how unpredictable a signal is; Luppi et al., 2019) or “small-worldeness” (which allows for a cost-efficient network organization; Uehara et al., 2014) are also affected in reduced consciousness. We also remain mindful of the fact that anticorrelations can still result by fMRI preprocessing steps, as described above, or from changes in the hemodynamics in some regions or even from time delays that produce phase lags. The DAN, for instance, was found to precede DMN deactivation by up to hundreds of milliseconds (Kucyi et al., 2020; Raccah et al., 2018). One possible explanation for that is that the anticorrelations are driven by sequences of spontaneous neuronal population events across the DMN and antagonistic systems that involve interregional temporal delays (Kucyi et al., 2020). The observed zero-lag anticorrelations could therefore be due to “blurring” of such delays, meaning anticorrelations could be the result of transient events that are comprised of spontaneous activations that are systematically coupled to subsequent DMN deactivations, as observed in transient coactivation patterns at rest (Karahanoğlu & Van De Ville, 2015; X. Liu & Duyn, 2013). However, this hypothesis requires further investigation. These issues keep our hypothesis about anticorrelations being a metric of consciousness in check and call for experimental and mechanistic explanations.

Collectively, we consider that FC anticorrelations are an essential ingredient for conscious mental activity and might work as another marker of preserved consciousness. We, therefore, predict that if the brain is not in a state to host the physiological prior of inhibitory activity, then most likely the individual does not entertain subjective experience. This stance opens new avenues for the understating and treatment of clinical cases of consciousness alternations by targeting anticorrelations specifically as the outcome measure, like it has been shown using neurofeedback (Bauer et al., 2020) or meditation (Bauer et al., 2019) for psychiatric disorders. We think that the introduction of yet another metric is justified by the difference this metric makes and the pragmatic issues it addresses (Demertzi et al., 2017). Hence, we align with the view that, when searching for consciousness, accumulative evidence stemming from multiple nonoverlapping assessments with different modalities needs to be applied (Seth et al., 2008)—ideally, those that receive theoretical framing in order to account for the mechanistic explanations of the metric at hand. What this view of the anticorrelations essentially offers is a link between the level of neuronal microcircuitry and the computational level, which starts gaining support when attempting to describe how consciousness happens (Changeux, 2017).

## CONCLUSIONS

We suggest that the FC anticorrelations emerge thanks to local and global neural inhibitory activity, which leads to variant spatiotemporal configurations. Such rich network organization was previously shown to characterize typical conscious conditions, while simpler interregional connectivity was most frequently seen in anesthetized states and states of low reportability (Barttfeld et al., 2014; Demertzi et al., 2019; Huang et al., 2020). We eventually invite researchers to view anticorrelations as the physiological expression of segregation via neural inhibition, which can help us not only comprehend how consciousness happens, but also conceptualize and design effective interventions for treating consciousness disorders in which anticorrelations seem particularly affected.

## ACKNOWLEDGMENTS

We would like to thank Mr. Fort Larry, M.A., for proofreading the manuscript for grammar and syntax.

## AUTHOR CONTRIBUTIONS

Athena Demertzi: Conceptualization; Investigation; Project administration; Resources; Writing – original draft; Writing – review & editing. Aaron Kucyi: Investigation; Resources; Validation. Adrián Ponce-Alvarez: Methodology; Validation; Visualization; Writing – review & editing. Georgios A. Keliris: Investigation; Methodology; Resources; Validation. Susan Whitfield-Gabrieli: Resources; Supervision; Validation. Gustavo Deco: Methodology; Resources; Supervision; Validation; Visualization.

## FUNDING INFORMATION

Athena Demertzi, Fonds De La Recherche Scientifique - FNRS (https://dx.doi.org/10.13039/501100002661).

## TECHNICAL TERMS

Functional anticorrelations: Negative functional connectivity that certain brain regions show with regard to the positive connectivity of other regions as observed in zero-lag correlations of BOLD activity.
Global signal: BOLD signal time course averaged across all brain voxels.
Neural inhibition: A physiological mechanism that helps sustain balanced cortical activity by orchestrating the coordination between excitatory pyramidal neurons and inhibitory nonpyramidal cells.
Network metastability: The ability of network transitioning among variant functional states over time.
Gain response function: A model parameter that can be set to different values across brain regions that impose various levels of excitability.
Ignition: The ability of a given brain area to propagate feed-forward and recurrent neuronal activity to other regions.
Neuronal bistability: The tendency of cortical neurons to fall into a silent period (down state) after an initial activation.
Segregation: The brain’s capacity to show selectivity in the way brain areas are functionally connected.
